# The Role of Ontogeny in the Evolution of Human Cooperation

**DOI:** 10.1007/s12110-017-9291-1

**Published:** 2017-05-18

**Authors:** Michael Tomasello, Ivan Gonzalez-Cabrera

**Affiliations:** 1 Max Planck Institute for Evolutionary Anthropology, 04105 Leipzig, Germany; 2 Department of Psychology and Neuroscience, Duke University, Durham, NC USA; 3 Konrad Lorenz Institute for Evolution and Cognition Research, Klosterneuburg, Austria

**Keywords:** Human cooperation, Shared intentionality, Cooperative breeding, Evolution of human ontogeny, Collaborative foraging

## Abstract

To explain the evolutionary emergence of uniquely human skills and motivations for cooperation, Tomasello et al. (2012, in *Current Anthropology* 53(6):673–92) proposed the interdependence hypothesis. The key adaptive context in this account was the obligate collaborative foraging of early human adults. Hawkes (2014, in *Human Nature* 25(1):28–48), following Hrdy (*Mothers and Others*, Harvard University Press, 2009), provided an alternative account for the emergence of uniquely human cooperative skills in which the key was early human infants’ attempts to solicit care and attention from adults in a cooperative breeding context. Here we attempt to reconcile these two accounts. Our composite account accepts Hrdy’s and Hawkes’s contention that the extremely early emergence of human infants’ cooperative skills suggests an important role for cooperative breeding as adaptive context, perhaps in early *Homo*. But our account also insists that human cooperation goes well beyond these nascent skills to include such things as the communicative and cultural conventions, norms, and institutions created by later *Homo* and early modern humans to deal with adult problems of social coordination. As part of this account we hypothesize how each of the main stages of human ontogeny (infancy, childhood, adolescence) was transformed during evolution both by infants’ cooperative skills “migrating up” in age and by adults’ cooperative skills “migrating down” in age.

Many of the most distinctive cognitive and social differences between humans and their nearest primate relatives derive from humans’ unique skills and motivations for cooperation. Tomasello et al. (2012) summarize these differences and hypothesize a two-step evolutionary sequence—from small-scale collaboration to culture-level cooperation—to explain their emergence in the human lineage. Hawkes (2014) does not dispute this general account, but, following Hrdy (2009), she emphasizes a subset of humans’ unique skills and motivations for cooperation and so hypothesizes an alternative evolutionary scenario focusing on the role of cooperative breeding, especially the unique demands it places on infants. Our attempt here is to synthesize these two evolutionary scenarios into one account that is more accurate and comprehensive than either alone.

## The Interdependence Hypothesis

To account for the emergence of uniquely human forms of cooperation, Tomasello et al. (2012) propose the interdependence hypothesis. Following Roberts (2005), the evolutionary logic of the hypothesis is that in highly social species individuals depend on one another for various aspects of their fitness. A primate individual’s most important mating partner, coalition partner, or, in some cases, foraging partner, are important to their fitness. Following the same logic as kin selection, there is thus an evolutionary benefit for helping these partners, and so the individual should invest, to some degree, in their fitness. There is obviously a mathematics to this investment determining the maximally beneficial level, just as there is in kin selection. And just as there is in kin selection, there is potentially a motivation to free ride—to let others help my kin or my valued partners—but often an incentive to do the helping oneself to make sure it gets done. Thus, interdependence means that individuals in social groups are constantly, to some degree, investing in the fitness of their group mates.

For the first step in the account, the proposal is that species-unique forms of human cooperation, and the underlying psychology supporting them, first emerged in the context of early humans’ shift to obligate collaborative foraging. The process very likely began around 2 million years ago with the emergence of the genus *Homo* and was consolidated by around 400,000 years ago with the emergence of *Homo heidelbergensis* (Stiner 2013). In terms of cognition, the interdependence hypothesis holds that humans’ unique cognitive skills first emerged to solve problems of social coordination in the context of mutualistic collaboration (Tomasello 2014). Much evidence suggests that humans collaborate with a partner in unique ways; for example, they form together a joint goal, which then creates two interrelated roles aimed at the same end (Fletcher et al. 2012; Hamann et al. 2012). Unique forms of human cooperative communication—initially the uniquely human communicative gestures of pointing and pantomiming—arose as means for coordinating such mutualistic activities (Tomasello 2008). Such cooperative communication depends on joint attention to external referents and the ability to recursively “read” the intentional states of others. In terms of sociality, early humans engaged in a kind of cooperativization of competition resulting in a sense of fairness in dividing the spoils of collaborative efforts in mutually satisfactory ways (excluding underserving free riders in the process). An important dimension of this process was partner choice and control (and the resulting social selection): individuals who could not peaceably divide the spoils of a collaborative effort with a partner (or otherwise cooperate) would not be selected as a partner in the future. Early human collaborative partners knew that each of them had to play their role in mutually known ways for their joint success, leading to processes of social evaluation and, eventually, moral judgment.

The second evolutionary step of the interdependence hypothesis proposes that all of this got scaled up as modern humans began forming cultural groups beginning some 150,000 years ago. All the members of a modern human cultural group understood themselves to be interdependent with their group mates for survival, including in the context of competition with other groups, and indeed members of other groups were thought of as free riders or competitors not deserving of any of the fruits of their collective labor. In this context, modern humans evolved, cognitively, the ability to create collective, group-minded cultural structures such as linguistic conventions, social norms, and cultural institutions to coordinate and regulate their group life.

Much of the evidence that Tomasello et al. (2012) cite for this account derives from experimental studies comparing the behavior of great apes and human children of various ages. Many of the important aspects of human uniqueness emerge during human infancy and early childhood, especially the uniquely human versions of collaboration, joint attention, cooperative communication, and resource sharing. Hawkes (2012, 2014) adopts the perspective of human life history theory and asks why these uniquely human cognitive and social abilities should emerge at such a young age. If it’s all about collaborative foraging and intergroup competition, why do infants need unique skills? In their response, Tomasello et al. (2012) merely point out that many skills important for adults begin their ontogenetic trajectories earlier in development because they require time for maturation, learning, and other forms of preparation. But further consideration deems this an inadequate response, as children develop the skills many years before they would be needed for their adult functions, with a long period of early and middle childhood in between. To date, then, the question of early ontogenetic emergence has not been answered from the point of view of the interdependence hypothesis.

## The Cooperative Breeding Hypothesis

For other extant apes, childcare is provided almost exclusively by the mother, and mother and infant are in almost constant bodily contact. But for humans, childcare is also provided by fathers, grandparents, and others in the social group, on average across many different cultures in the contemporary world at the rate of about 50% after early infancy. Hrdy (1999, 2005, 2009) identified this pattern as an instance of cooperative breeding—broadly defined to include all systems in which alloparents as well as parent help care for and protect offspring—which has a number of somewhat different manifestations across species. Prototypically, many bird species—and among primates the callitrichidae (marmosets and tamarins)—practice cooperative breeding, with helpers doing such things as provisioning and transporting infants. The evolutionary bases of cooperative breeding systems often involve kin selection, and sometimes individuals are punished by others in the group if they do not help (so-called pay to stay).^1^ Human mothers benefit from childrearing help in many ways, not least of which is the ability to care for more offspring.

Hrdy (1999, 2009) notes that in cooperative breeding species helpers must have at least some kind of prosocial motivation, even if their goal to help others is grounded evolutionarily in kin selection or proximately in a desire to avoid punishment. In the human case, kin selection is clearly not the whole story, as helpers are often other unrelated adults, and punishing non-helpers does not seem to play an important role. In this case, then, helpers should evolve especially strong prosocial motivations. Burkart and van Schaik (2010) surveyed observational and experimental data on cooperative breeding species across a variety of different taxa and concluded that indeed cooperative breeding leads to, among other things, more prosocial motivations. Hawkes (2014) proposes some additional hypotheses. Following Hrdy, she focuses on the implications of a human-like cooperative breeding system not just for the helpers but for the infants themselves (what might be called the “development plus social selection model” for the effects of the human cooperative breeding system on ontogeny; Hrdy 2016). Human infants face a new challenge as they grow up with a mother who, unlike other primate mothers, leaves them for some periods of time with other adults. The infants must then compete with siblings and other infants and young children for the attention and care of their mother, and, in addition, the attention and care of less-familiar caregivers. Because cooperative breeding enables human mothers to have more closely spaced offspring, there is, presumably, heightened sibling competition.

The human cooperative breeding system thus sets up a selective context in which those infants do best who can discern the thoughts and moods of caregivers and solicit help and care best through various kinds of emotional and referential communication. The outcome is that not only have humans been selected to be especially prosocial as helpers in a cooperative breeding system, the human infancy period has come under special selection pressures for various skills of social cognition and communication. Both Hrdy and Hawkes single out for special attention various skills of shared intentionality, which have been shown to be unique to the human species and of special importance in the evolution of cooperation and culture (e.g., Tomasello et al. 2005, 2012). The foundational skills, which emerge in human infancy and early childhood, concern such things as joint attention (the intersubjective sharing of experience) and cooperative communication via gestures such as pointing and, ultimately, language. The infant adaptations are special in this account because they are seen as ontogenetic adaptations for the infancy period, not as deferred adaptations whose original function was to promote the fitness of adults. This is clearly a different hypothesis from that of Tomasello et al. (2012), who have posited that humans’ unique skills and motivations of shared intentionality are adaptations mainly for adults’ uniquely cooperative forms of social life, especially as manifested in cooperative foraging and other cultural lifeways.

Tomasello et al. (2012) acknowledged a role for cooperative breeding in the evolution of humans’ lesser aggression and dominance relative to other apes, and greater tolerance around food. But they did not agree with Hrdy and Hawkes that shared intentionality—at least in its foundational aspects—is an ontogenetic adaptation enabling infants to better navigate the special social world created by a cooperative breeding system. In particular, they argued that cooperative breeding would not seem to create the kinds of cognitive challenges that would lead beyond the relatively simple kinds of things that infants do to capture attention and solicit care to such cognitively complex things as social conventions, norms, and institutions (including conventional languages). For the evolution of these things we need the kinds of cognitive challenges that adults face, perhaps in the context of obligate collaborative foraging and, later, cultural life.

The result of all of these considerations is that the interdependence hypothesis has a weakness in that it has no ready explanation for the early ontogenetic emergence of skills of shared intentionality, and the cooperative breeding hypothesis has a weakness in that it has no ready explanation for how humans go beyond joint attention and gestural communication to the complex cognition underlying human cultural life.

## A Composite Account

Our proposal here is that a comprehensive account of the evolution of human cooperation—and the psychological skills and motivations of shared intentionality that go with it—requires insights from both the interdependence and the cooperative breeding hypotheses. Following Hrdy and Hawkes, what we need is an evolutionary scenario that takes into account human life history, that is, the adaptations that have emerged to deal with the challenges of each ontogenetic period on its own terms. In addition, we need a scenario in which adaptations originally associated with one ontogenetic period may be extended to different ontogenetic periods, with cascading effects as they interact with existing developmental pathways in novel ways.

We focus here on Tomasello et al.’s (2012) first step in the evolution of uniquely human cooperation, a period during which early humans (from *Homo erectus* to *Homo heidelbergensis*) began cooperating in new ways but did not yet have cultural organization. (Later we speculate briefly about the ontogeny of modern humans, *Homo sapiens*, after the emergence of culture.) The proposal is that these new ways of cooperating involved both collaborative foraging and cooperative breeding, which would have been complementary components in an overall more cooperative lifestyle. We leave open the original selective context for cooperative breeding—which presumably went along with the emergence of human pair bonding (Chapais 2008)—but only claim that at some point these two forms of ultra-cooperative interaction were mutually supportive.

### Evolution and Ontogeny

A central insight of evolutionary developmental biology (evo-devo; e.g., West-Eberhard 2003) is that many, if not most, significant evolutionary changes result from changes in ontogenetic timing. That is, if an adaptation emerges earlier or later in ontogeny, or takes shorter or longer to develop, in a modern population as compared with an ancestral population, the result can be huge changes in the phenotype because of the interaction of this change with existing developmental pathways. For example, when over evolutionary time juvenile characteristics come to be extended into adulthood in a pattern of neoteny, the effects may extend well beyond that characteristic itself (e.g., childhood curiosity combines with adult cognitive and social skills to result in science). Or if it were the case that running was originally an adaptation for adults stalking prey, if it extended to an earlier period in ontogeny, it might now be adaptive for children outrunning predators.

The way such changes happen are strictly Darwinian, but in a developmental context. Thus, many changes in developmental timing are a simple result of the fact that because of random variation an adaptation emerges in some individuals a bit earlier or a bit later than the norm, or the adaptation takes shorter or longer to develop than is typical. If there is a fitness benefit for individuals at the extreme—those in whom the adaptation emerges earliest or latest—then a change in timing should occur. The fitness benefit is first and foremost about the particular developmental periods involved, but it may extend to later developmental periods if, for example, skills developed at one period are especially good preparation for challenges to be faced at some later period. Any time there is a potential change in developmental timing, there are “developmental constraints” that must be overcome, in the sense that the change in timing of one system cannot disrupt already existing systems inordinately. Organisms must be fit at each step in ontogeny independently—and this cannot be disrupted—if they are to live to pass on their genes, with the greatest fitness benefits going to those who live long and fecund lives.

In this context, the interdependence hypothesis can be recast as follows: adaptations for shared intentionality that were beneficial in adult activities (i.e., collaborative foraging) “migrated down” in ontogeny because they benefited immature individuals in their activities as well. This migration process, in turn, also benefited affected adults because developing skills earlier enabled extra preparation and practice for collaborative activities in adulthood. In the same spirit, the cooperative breeding hypothesis can be recast as follows: adaptations for shared intentionality that were beneficial to infants and young children in a cooperative breeding context “migrated up” in ontogeny because they benefited these children in their later collaborative and communicative activities as well. These individuals were then better adapted, in some sense by chance, for adult activities relying on collaboration and communication.

Our proposal is that in fact both of these processes occurred. That is, we propose that human cooperative breeding and collaborative forms of subsistence coevolved because mothers who received help rearing their young could be much more productive in collaborative foraging (and the helpers would share in the fruits of her labor). This combined hypothesis would explain both why skills of shared intentionality first emerge in contemporary human ontogeny in infants and young children—that is, as ontogenetic adaptations for infancy in a human-like cooperative breeding situation—and why contemporary humans have the complex cognitive skills for social and cultural coordination that they have—that is, as these ontogenetic adaptations extended to older children and adults and interacted with other systems for solving adult problems of social and cultural coordination (and perhaps set the stage for new adaptations for these older individuals as well) in ways that increased adult fitness.

Our composite account thus consists of both bottom-up and top-down processes of natural selection operating within ontogeny. Bottom-up processes are those in which an ontogenetic adaptation migrates up and—by chance, as it were—increases the fitness of the adult individuals who possess it, a kind of ontogenetic preadaptation for adulthood. But, of course, if there is a fitness benefit for adults who possess the competency, then this creates a kind of ontogenetic top-down selective pressure as well for younger individuals to develop the competency as a deferred adaptation (assuming that a longer ontogeny, beginning earlier, is somehow advantageous for adults as preparation for the “real thing”). With this background, of special importance in the current account are three relatively distinct periods of ontogeny, each with its own set of adaptive challenges: (*i*) infancy and toddlerhood (before weaning, approximately 0–3 years); (*ii*) early and middle childhood (weaning to sexual maturity, approximately 4–12 years); and (*iii*) adolescence and adulthood (sexual maturity, approximately 13 years and beyond).

### Infancy and Toddlerhood (Pre-Weaning; 0–3 years)

Hrdy’s (2005, 2009) basic proposal is that in a cooperative breeding context, in which mothers had much help, they had babies at more closely spaced birth intervals than did other apes. Mothers were thus forced to divide their care and attention to offspring more than other apes, and indeed to relinquish physical contact and responsibility to other adults to care for their offspring (in a way that other apes do not systematically do). In her “development and social selection” corollary hypothesis, children of all ages would then have had to compete more strenuously for adult care and attention, and they would have had to learn to deal with different adults more flexibly (see Sulloway 1996 on the important role of sibling competition in human development).

Neither Hrdy nor Hawkes goes into much detail about the uniquely human skills and motivations of shared intentionality involved, but there are several. First, at around six weeks of age infants begin smiling at others engagingly and from a distance, followed some weeks later by laughing at others engagingly and from a distance. Although one may identify some precursor behaviors in infant chimpanzees when they interact with humans (e.g., Bard 2012), they do not smile and laugh at others from a distance in their natural social development with mothers or other conspecifics. From around 2 months of age, human infants begin to engage in the kinds of positive-emotion-sharing interactions with adults made possible by these unique social behaviors in turn-taking sequences that some researchers have dubbed “protoconversations” (e.g., Trevarthen 1979; see also Reddy 2015; Rochat 2009). The adaptive function of these kinds of infant behaviors is not attachment; attachment has its own mechanisms with their own evolutionary history, presumably functioning mainly to ensure protection, reaching back well before the emergence of primates (Bowlby 1969). Rather, the adaptive function of these unique infant behaviors is, plausibly, to secure adult care, feeding, and other forms of attention by sharing positive emotional states, which are reinforcing to both parties.

Tomasello et al. (2005) propose that human infants’ skills and motivations for emotion sharing in protoconversation are transformed, beginning at around 9 months of age, into a suite of uniquely human skills and motivations for shared intentionality. At around 9 months of age, human infants are beginning to understand others as intentional agents (with goals and perceptions that guide their behavior) in the same basic way as do other great apes, although, interestingly, human infants seem to develop these skills at a somewhat earlier age (Wobber et al. 2013). What happens, then, is that the sharing of emotions extends to the sharing of attention and interest in external objects and events—behaviors in which great ape infants never engage (see Tomasello and Carpenter 2005; Tomonaga et al. 2004)—via a synergistic combination of uniquely human emotion sharing and great ape–wide cognitive skills for understanding the intentionality of others. These joint attentional behaviors are not just following the gaze direction of others—which great apes do from an early age as well (e.g., Tomasello et al. 2001)—rather they involve more active acts such as showing and offering objects to others, with an expectation that this will lead to a positive sharing of emotions about these objects and/or their associated events. In the analysis of Bruner (1983; see also Tomasello 1995), these unique forms of social interaction involve a recursive cognitive process in which each expects the other to expect them to expect . . . (and so on). Importantly, Bakeman and Adamson (1984) found that infants engage in joint attention much more readily with adults than with infant peers, suggesting adults as special targets. And in an experimental study with adults, Wolf et al. (2015) found that subjects showed more signs of social bonding with a partner after they had engaged with him in joint attention.

An especially important outgrowth of infants’ joint attentional behaviors is cooperative communication as expressed prelinguistically in the uniquely human gestures of pointing and pantomiming. Whereas great apes will sometimes point for humans (not conspecifics) imperatively, for things they want (e.g., Leavens and Hopkins 1998), from around their first birthday human infants point expressively to simply share with an adult their enthusiasm for some object or event (see Tomasello et al. 2007 for a review). They typically do this dozens of times per day every day (Matthews et al. 2012), and they do it beginning at about the same age in all human cultures investigated (Callaghan et al. 2011; Salomo and Liszkowski 2013). In what has been called the nine-month revolution, then, human infants begin to be highly motivated to share attention to and interest in things with adults—indeed, to initiate such shared attention and interest by offering, showing, and pointing referentially to external events and entities. Their motive is not just to get the other to look at the object or event, but rather that the adult share a sense of interest and enthusiasm for it (Liszkowski et al. 2006). They also, and also uniquely, use the pointing gesture to inform adults of things to help them—for example, indicating the location of a sought-for object (Liszkowski et al. 2008). Importantly, Ninio (2016) found that contemporary human infants use their communicative skills much more readily and often with adult partners rather than with peers (see also Stoeber et al. 2017).

The motive to help others is already present in great apes (e.g., Warneken et al. 2007; Melis et al. 2011). But human infants help others, especially adults, in some unique ways. In addition to helping them by informing them of things with the pointing gesture, young human children seem to take the perspective of the other when engaging in such helping—for example, by providing extra help to those who have previously been harmed (Vaish et al. 2009), which apes do not do (Liebal et al. 2014). And, once again, although a properly controlled experimental study has not been done, comparing across studies one can infer that human infants help adults at a significantly higher rate than they help other infants (Warneken and Tomasello 2006; Hepach et al. 2016).

The proposal is thus, following Hrdy (2005, 2009), that these uniquely human infant behaviors evolved in the context of sibling and peer competition for adult care and attention. Adults are rewarded by sharing emotions, interest in, and attention to objects and events with infants, and so infants compete to provide adults with these rewards. Adults thus engage in a process of social selection (West-Eberhard 1979) in which the infants that are best able to engage them get the most attention. Following Hrdy (2005, 2009, 2016), as elaborated by Hawkes (2012, 2014), then, we may see the earliest ontogenetic emergence of skills and motivations for shared intentionality as adaptations for sibling/peer competition for securing adult attention. Note that in this hypothesis, it must be infants who lead the way in skills and motivations for joint attention. In the early evolutionary steps, they must have used joint attentional skills to “hijack” already existing adult emotional and bonding mechanisms for their own ends. Adult skills and motivations for shared intentionality would have then followed as these skills and motivations also provided fitness benefits to individuals at older ages (see Table 1 for a summary).

**Table 1:** Species-unique behaviors of human infants evolved to solicit care and attention (and/or bonding) from distracted adults in cooperative breeding context

Uniquely Human Infant Behavior	Function in Cooperative Breeding Context	Relevant Reference(s) to Apes and Human Infants
Intentional crying and imperative gestures	Requesting help from adults	Tomasello (2008) for a review
Helping and informative gestures	Offering help and information to adults	Bullinger et al. (2014); Liszkowski et al. (2008)
Social smiling and laughing at a distance	Recognition of and affiliation to recipient	Trevarthen (1979); Bard (2012)
Expressive gestures (e.g., pointing to share interest)	Aligning psychological states (joint attention) as way of bonding	Tomasello and Carpenter (2005); Wolf et al. (2015)

### Childhood (Weaning to Sexual Maturity; 4–12 years)

Human infants wean a year or two younger than other apes, at around three years of age, but then they, unlike other apes, depend on adults for provisioning, protection, and other care well into adolescence. During the still-dependent periods of early and middle childhood, then, human children are still engaged in a process of sibling/peer competition for adult care and attention. In addition, children in this age range are dependent on adults for learning many necessary things about their shared world and how best to accomplish things in it. One way children gain the needed adult care and attention is by making themselves especially useful to adults (ingratiating themselves with adults) by helping them with various tasks. Kramer (2005) has thus emphasized that human children often help their parents and caregivers in various ways, and so cooperative breeding actually fosters a two-way interdependence between adults and youngsters.

But, in addition, early and middle childhood also ushers in a whole new social world for children: the world of peers. Interactions with adults are inherently asymmetrical because adults possess all of the power, competence, and knowledge, which infants and young children respect and defer to. But interactions with peers are more symmetrical since peers have no special power, competence, or knowledge; they must be dealt with on an equal footing. The child cannot rely on or defer to a peer partner as she can an adult partner, and so things must be worked out. On the one hand, being too shy and unassertive will result in being taken advantage of, but, on the other hand, attempting to be too dominant might very well end up in ostracism. With specific reference to moral development—as uniquely human internalized cooperative tendencies—Piaget (1932) describes childhood as inhabiting two distinct social worlds, one based on authority (with adults) and the other based on reciprocity (with peers).

The peer world is the world the individual will inhabit as an adult. This is a world in which things must be worked out among more-or-less equal partners. In this sense, children’s adaptations for interacting with peers during early and middle childhood are very likely deferred adaptations, whose adaptive significance lies in the ways in which they prepare children for adulthood. During early and middle childhood children learn to make decisions with others—for example, about where to go or what to play (Köymen et al. 2014). They learn to treat others fairly—for example, by sharing resources in a mutually satisfactory manner (Hamann et al. 2011). They learn to make and keep joint commitments, as they commit to doing something in the future and must keep it or risk reprisals (Gräfenhain et al. 2009). All of this is cemented in human development by a unique tendency (i.e., unique among apes): young children, but not other apes, are concerned about others’ evaluations of their cooperative tendencies, and they adjust their behavior based on their prediction of how others will assess it, so-called impression management (Engelmann et al. 2012). And thus children do not have to try out things and get feedback on how others view them; rather they self-regulate their own behavior by anticipating how others will assess it.

The proposal is that early human children who developed more and better skills of shared intentionality fared better in this new world of peer interaction. Hence the infants’ ontogenetic adaptation for shared intentionality in joint attentional and communicative interactions with adults “migrated up.” As a crucial part of the process, in early and middle childhood a new process emerged of social selection involving not adults but peers. Perhaps often in the protected context of play, but sometimes in more serious contexts as well, young children must cooperate with others or suffer the consequences. They know this, and so they develop a kind of cooperative identity—simultaneously a social persona and an internalized sense of self—for social interactions with peers. Arguably, this does not and cannot develop out of interactions with adults, who do not cooperate but rather legislate. If these kinds of childhood interactions are conceived as preparations for adulthood, two consequences ensue. On the one hand, one’s interactions with same-gender peers are crucial in developing a cooperative identity for engaging in various collaborative tasks. And, on the other hand, despite the pronounced gender segregation of the social interactions during middle childhood, this will very likely have effects on the judgments of individuals of the opposite gender as well and thus, in a community of mostly familiar peers, will have effects for one’s sexual prospects in adulthood.

The outcome is the two social worlds of childhood. During early and middle childhood, there is social selection from three sources: (*i*) adults selectively dispense attention and resources to children whom they like and who help them; (*ii*) same-gender peers prefer and selectively interact with good cooperators; and (*iii*) opposite-gender peers begin selecting for mates with good prospects and status in the social group. But, as Tomasello (2016) has argued, it is not just that children of this age are being controlled by “them,” but rather they have internalized the collaborative role standards used by everyone as coming from “us,” and so they develop a genuine sense of cooperative identity that guides everything they do. Skills and motivations of shared intentionality fostered during infancy give individuals an adaptive advantage in these interactions, and once that advantage is established, a top-down process ensues in which the ontogeny of infants’ skills can also have deferred adaptive benefits at the later stage of childhood.

### Adolescence/Adulthood (Sexual Maturity; 13+ Years)

During adolescence, things begin to get real. Whereas many of the cooperative interactions of childhood take place in playful contexts, during adolescence and adulthood one must collaborate with one’s peers for basic subsistence (and, again, with ramifications for one’s attractiveness as a sexual partner).

In the scenario for early humans that we are envisioning here, there would have been an initial period in which they were mostly individual foragers, like all apes, but with a few pockets of collaboration. Like chimpanzees and bonobos, perhaps, they would have hunted in small groups for monkeys and other small mammals. If there were now selective pressures for collaborative foraging—Tomasello et al. (2012) point out a period during the early Pleistocene in which there was a rapid radiation of terrestrial monkeys, who might have outcompeted early humans for their preferred fruits—then those individuals who were best able to collaborate with their peers would have had an adaptive advantage. It is in this context that we may speculate that those individuals who had already developed especially powerful skills and motivations of shared intentionality, beginning in infancy and continuing throughout childhood, would already have a special stock of skills needed for new forms of collaborative foraging. The outcome is an ontogenetic preadaptation: an ontogenetic adaptation selected for at an early developmental period that turns out to be useful for a later development period, including especially adulthood during which individuals actually pass on their genes.

We may thus picture a kind of pincer movement of bottom-up and top-down selective pressures. There are pressures for infants’ ontogenetic adaptations for joint attention, cooperative communication, and helping, to “migrate up” because they are useful to children and adults in the context of ecological pressures for collaboration and collaborative foraging. And these ecological pressures for collaborative adults could easily begin to “migrate down,” at least into early and middle childhood when children are learning to collaborate with peers in various simple, sometimes playful contexts, which prepares them for adult collaboration in serious contexts. We may thus imagine early humans gradually evolving a more cooperative lifestyle in general based mainly on cooperative breeding and cooperative foraging, which fit together like hand in glove. Each of these new and unique ways of surviving made for an especially cooperative ape, each with its primary focus at a different ontogenetic period but with possibilities for “migration” either up or down, initiating all kinds of synergistic effects.

### Some Notes about Modern Humans and Culture

As just a brief note: with the emergence of modern humans and culture, some new forces come into play. These are not our focus here because they come rather late in the process. But we may at least note two extremely important new developments in the current context: (1) active adult instruction of children in the ways of the culture, often embodied in explicit cultural norms, and (2) child conformity to instruction and norms based not just on prudence but on a kind of group-mindedness in which the good of the group is a basic concern alongside other basic concerns. Indeed, we think that the increase in behavioral and cultural complexity from anatomically modern humans to behaviorally and culturally modern humans several tens of thousands of years later is explained by the emergence of human group-mindedness as a new layer of “top-down” influences on human ontogeny.

Complex social groups rely on economic specialization, division of labor, and social norms and conventions. Norms and conventions require not only increased common cultural ground but also extending trust to all in the group. As economic and demographic complexity increases, cooperation gets more challenging and reliant on delayed returns in a different currency, often through a third party (Sterelny 2012). As a result, in large and culturally complex societies, successful cooperation and coordination rely heavily upon trusting strangers (Seabright 2004), based not just on individual experience but also on cultural norms and values, especially those that signal group identity. The key point is that modern human adults began to construct a wide variety of cultural conventions, norms, and institutions to solve adult problems of cooperation and coordination, which were transmitted to young children as injunctions to conform. These may just be seen as a new layer of “top-down” influences on human ontogeny, in the sense that these cultural creations are adapted to adult concerns, and they trickle down to children as they prepare to become adults (i.e., following these norms is adaptive not only during childhood, but also as preparation for adulthood). This new group-mindedness also manifests in a new concern with distinguishing in-group members from out-group members in the context of group competition and cultural group selection (Henrich 2015).

By the time of modern humans, then, we have a hyper-cooperative lifestyle based on cooperative breeding, cooperative foraging, and cooperative sharing of cultural information (Sterelny 2012), with ontogenetic adaptations migrating both up and down. In modern humans, there were additional and special “top-down” selective pressures in which adults expected children to conform the culture’s way of doing things in order to further the interests of the group.

## Conclusion

The study of human life history has focused almost exclusively on such things as weaning age, reproductive age, longevity, and other basic processes of survival and reproduction. The study of human psychological development, in contrast, has focused very little on the evolutionary bases of different periods of ontogeny. A full understanding of how human beings have come to be as they are requires an understanding of both human evolution and human ontogeny with regard to a variety of life history variables and psychological competencies as well.

Our specific conclusion here is that to explain the many and various unique forms of human cooperation requires both the cooperative breeding hypothesis, positing a unique selective environment for fostering the most basic skills and motivations of shared intentionality during infancy, and the interdependence hypothesis, positing a critical role for the obligate collaborative foraging of adults as a selective environment favoring individuals who could engage in various forms of complex social coordination and communication. The cooperative breeding hypothesis by itself cannot account for the many complex cognitive developments associated with human cooperation, but the interdependence hypothesis by itself cannot account for the early emergence of skills and motivations for shared intentionality during human infancy. A composite account is needed, and our attempt here has been to provide the beginnings of such an account. Empirical support for our composite account is of course not straightforward. But we have provided at least some evidence here—and would hope to be able to provide more in the future—that (*i*) human infants’ initial skills of shared intentionality are mostly adapted for interaction not with peers but with adults; (*ii*) infants’ engaging with adults in acts of shared intentionality is inherently pleasurable for both parties and so, presumably, serves to enhance their relationship; and (*iii*) older children begin to develop further skills and motivations for engaging in acts of shared intentionality with peers.

Although in some sense implicit in evo-devo accounts, our analysis here has also highlighted in a new way the process by which ontogenetic adaptations geared toward a particular developmental period may be extended either up or down in age as a natural consequence of random variation in age of onset and selective pressures present during adjacent developmental periods. Cooperative breeding thus generates during infancy ontogenetic preadaptations for cooperation that later prove useful for older children and adults, and interdependent collaboration during adulthood creates pressure for ever-younger children to begin acquiring and practicing their collaborative skills. For the full story we need both phylogeny and ontogeny and how they relate.

## Footnotes

1 The evolution of cooperative breeding in the human lineage is estimated to have happened around 2.0–1.8 million years ago with *Homo erectus* (see Burkart and van Schaik 2010). In other cooperative breeding species, pressures that lead to cooperative breeding include predation risk, environmental unpredictability, and those associated with the colonization of new habitats (Clutton-Brock 2006; Hrdy 2005, 2009; Isler and van Schaik 2012). These selective pressures likely played some role in the evolution of cooperative breeding in the human lineage. For example, predation risk is expected to intensify with shifts toward more open habitats (Hart and Sussman 2005), and the most significant shift toward open landscapes occurred after 2.0 million years ago (Lee-Thorp, Sponheimer, and Luyt 2007). Moreover, conditions of increasing seasonality during the Pleistocene also led to environmental unpredictability (Potts 1998). Finally, early *Homo* is known to have colonized large areas outside Africa around the same period, which has been traditionally linked to the emergence of cooperative breeding (Burkart and van Schaik 2010; Hrdy 2005, 2009).
